# Shelf-Life Evaluation of Pork Loins as Influenced by the Application of Different Antimicrobial Interventions

**DOI:** 10.3390/foods11213464

**Published:** 2022-11-01

**Authors:** David A. Vargas, Sabrina E. Blandon, Oscar Sarasty, Andrea M. Osorio-Doblado, Markus F. Miller, Alejandro Echeverry

**Affiliations:** 1 Department of Animal and Food Sciences, Texas Tech University, Lubbock, TX 79409, USA; 2 Department of Agricultural and Applied Economics, Texas Tech University, Lubbock, TX 79409, USA

**Keywords:** indicator bacteria, linear regression analysis, 1,3-dibromo-5,5-dimethyl hydantoin, rhamnolipids, chlorine dioxide

## Abstract

The objective of the study was to determine the impact of antimicrobial interventions and refrigerated dark storage on the shelf-life of pork chops. Boneless pork loins (*n* = 36) were split and stored for 1, 14, 28, and 42 days at 2–4 °C after being treated with the following antimicrobials: water (WAT), Bovibrom 225 ppm (BB225), Bovibrom 500 ppm (BB500), Fit Fresh 3 ppm (FF3), or washing solution 750 ppm (WS750). After the end of dark storage, pork loins were further processed and sliced into chops, overwrapped in trays, and displayed for up to an additional 96 h in a retail case. Instrumental and visual color measurements as well as mesophilic and psychrotrophic aerobic bacteria, and lactic acid bacteria were measured. BB500 and FF3 performed better in inhibiting the growth of indicator bacteria under 6 logs; however, FF3 presented the best stability for color during storage. Principal component analysis clustered initial dark storage days with a* and chroma while % discoloration, hue, b* and microorganisms where clustered with longer dark storage times. In general, treatment FF3 presented the best performance, both in inhibiting microbial growth and maintaining the stability of color, thus increasing the shelf-life of pork loins.

## 1. Introduction

Pork is one of the most common consumed meats around the world [1,2]. Approximately 37% of all meat consumed worldwide is pork. Based on the report by the United States Department of Agriculture (USDA) Livestock and Poultry: World Markets and Trade, a total of 111.1 million metric tons of pork were consumed in 2018 [3]. However, this consumption had a drastic change that same year when Asia was hit with the African Swine Fever (ASF) and therefore there was a shortage of pork globally. Many ASF outbreaks throughout the years have been linked to improper handling of pork products and infected pigs with ASF [4]. Global consumption of pork meat is projected to double by 2050, driven largely by population growth and increase in income [5,6].

According to the Food and Agriculture Organization of the United Nations (FAO), in 2018 the total pork production was 120.5 million metric tons with China being the leader of the top countries for pork production with 48% but suffering loss by the ASF and dropping to 39% of the total production [7,8]. China’s Ministry of Agriculture and Rural Affairs reported there was a decrease in the stock of hogs from 320.8 million to 190.9 million [9]. Pork production was followed by European Union, United Sates, and Brazil with 21%, 11%, and 3%, respectively [10].

Usually, consumers take into consideration key aspects such as safety and quality when purchasing pork meat. Many microbial and physico-chemical changes can occur in food products according to their composition and properties [11]. Microbial contamination can be one of the most detrimental factors that can affect not just the product quality but also the consumers’ health. Many foodborne illnesses are attributed to the consumption of contaminated pork. As an example, *Salmonella* spp. is one of the most frequent foodborne pathogens with a 12.8% estimated percentage of foodborne illnesses in pork for 2019 report in the United States [12]. Recent studies show that retail pork has an overall prevalence of *Salmonella* spp. ranging from 4% to 73.1% [13]. Attribution of illness in the United States indicate that pork consumption is responsible for approximately 525,000 foodborne infections, 2900 hospitalizations, and 82 deaths each year [14]. In addition to *Salmonella* spp., other foodborne bacteria linked to pork also include *Yersinia enterocolitica*, and *Trichinella spiralis* as the most important known pathogens that cause serious foodborne illnesses [15,16].

To address and reduce the potential risk of contamination, many antimicrobial interventions have been implemented in pork processing facilities. Currently in the United States, hot water, chlorine, lactic acid or acetic acid are considered food grade antimicrobials approved to be applied to pork surfaces during processing [17]. They can be applied through hand-held sprayers or automated spray washers, but their efficacy will depend on several factors, including the spraying system used, spraying pressure, concentration, time, and temperature [18]. Despite many scientific studies demonstrating the effectiveness of interventions on the reduction in pathogens, there are many regulatory restrictions in place that limit the level of organic acids to be used [19]. Moreover, in addition to improving the safety of pork meat, the impact of antimicrobial agents on the overall quality and shelf life, two of the most important purchasing factors for consumers, must be explored.

Studies have shown that pork has a shorter shelf life than other type of meats. Fresh pork can only be preserved for up to six days with an adequate hygienic and temperature control [20,21]. Shelf life can be related to sensory characteristics of a product such as odor, flavor, and color. Meat deterioration can be identified by microbial spoilage, color loss, lipid oxidation, and water exudates [20]. This deterioration may cause off-odor and off-flavor development, changes in texture, and discoloration of meat [22]. Essentially, consumers determine food quality by product color and may respond differently to variations because it is one of the first and most determining sensory characteristics evaluated during retail display [23,24]. Retail consumers often develop assumptions about the product freshness, processing steps, and nutritional value based just on the color observed in the display refrigerators. Generally, consumers lean towards a dark-colored pork rather than light-colored pork. This can be correlated to the amount of marbling found in pork where highly marbled pork have a lighter pink color and the visible fat may be one of the strongest factors a consumer considers when purchasing pork [25].

Several instrumental methods are being used to monitor color in the meat industry, including the use of spectrophotometers or colorimeters. The most common way to evaluate objective measurements is using the CIELAB color system [25], where color is defined as a point in a three-dimensional space in relation to coordinates L* (lightness of the color), a* (wavelengths corresponding to colors from green to red), and b* (wavelengths corresponding to colors from blue to yellow) [26]. Instrumental or objective measurements are of great interest to the industry because of the consistent measures and speed that can be obtained [27]. However, a different method to assess pork color is through subjective or sensory evaluations. Because the perception of color is very dependent on the observer, it is important to know the value of relative objective measurements to the subjective judgment of acceptable color [28]. Pork color is difficult to assess due to the differences of color even within the same muscle [23]. For consumers, fresh pork meat is expected to have a uniform reddish pink color [28].

The purpose of this study was to determine the shelf-life, acceptability, and sensory characteristics of pork chops stored under prolonged refrigerated conditions before retail display after the application of multiple antimicrobials by measuring indicator bacteria and changes in color.

## 2. Materials and Methods

### 2.1. Sample Collection

Vacuum packaged boneless pork loins (*n* = 36) were purchased from a commercial processing plant and transported in a cooler covered with ice at 0–4 °C to the Gordon W. Davis Texas Tech University Meat Science Laboratory (Lubbock, Texas, TX, USA) within 4–5 h. Pork loins were placed in a box and stored under dark conditions (no light) at 0–4 °C and processed 24 h later. The samples collection was repeated three times between January to August of 2019.

### 2.2. Treatment Preparation

For each treatment, three to four liters of solution were prepared following label instructions or manufacturer equipment guidelines. Treatments included: cold water, Bovibrom 225 ppm (1,3-Dibromo-5,5-dimenthylhydantoin; prepared in a mixer provided by Passport Food Safety Solutions, West Des Moines, IA, USA), Bovibrom 500 ppm (prepared following the same steps as Bovibrom 225 ppm), Fit Fresh 3 ppm (chlorine dioxide; prepared following label instructions, Selective Micro Technologies, Dublin, OH, USA), and Natural Washing Solution 750 ppm (rhamnolipid, Jeneil Biosurfactant, Saukville, WI, USA).

### 2.3. Treatment Application

Pork loins were split into five sections of 8.90 cm in length and randomly assigned to one of the five treatments (*n* = 12 pork loin sections/treatment). Interventions were applied onto the pork loins for 30 s on each side using a handheld sprayer (Chapin 1-Gallon Plastic Tank Sprayer, Batavia, NY, USA; Flow rate: 5.98 ± 0.75 mL/s) ensuring to cover the entire loin surface. Treated sections were vacuum packaged after 10 min before intervention using Cryovac bags (Seales Air, Charlotte, NC, USA) and randomly assigned to one of the four dark storages periods (1, 14, 28, and 42 days) and refrigerated at temperatures around 0 to 4 °C.

### 2.4. Pork Chops Fabrication

At the end of each storage period, each section was further processed and sliced into four chops, 1.27 cm thickness each. Further, each chop was randomly assigned to one of three different retail cold storage periods (0 h, 48 h and 96 h) and one for color analysis in a retail case. Each chop was placed in polystyrene trays, overwrapped with an oxygen-permeable, polyvinyl chloride film, and displayed in a retail case under continuous fluorescent light at 0–4 °C. Trays with chops were repositioned every day from side to side and front to back in the retail case to reduce variability due to temperature and/or lighting intensity within the display case.

### 2.5. Swab Sample Collection and Processing

Pre-hydrated swabs (3M™, St. Paul, MN, USA) with 25 mL of buffer peptone water (BPW) were taken from both sides of the pork chop immediately after cutting the pork loin sections (0 h) and at the end of 48 h and 96 h under display cooler conditions. Swabs were taken to the ICFIE Food Microbiology Texas Tech University laboratory for microbiological analysis and homogenized in a bag mixer (Model 400 circulator, Sewars, West Sussex, UK) at 230 rpm for one minute. Then, samples were serially diluted in 9 mL BPW (Millipore Sigma, Danvers, MA, USA) tubes and plated in petrifilms (3M™, St. Paul, MN, USA) according to each microorganism. For Aerobic Plate Counts, the Association of Official Agricultural Chemists 990.12 (AOAC) official method was followed. Petrifilms were incubated for 48 ± 3 h at 35 ± 1 °C for mesophilic bacteria conditions (APC-M) and 72 ± 3 h at 20 ± 1 °C for psychrotrophic bacteria conditions (APC-P). For Coliforms and *Escherichia coli*, the AOAC 991.14 official method was used with an incubation of 48 ± 3 h at 35 ± 1 °C. For lactic acid bacteria (LAB), one mL of sample was inoculated on a Petri dish and pour plated with 20 mL of Mann-Rogosa-Sharpe Agar (MRS) in duplicates. Plates were placed in BD GasPak EZ Container Systems (Becton Dickinson and Company, Franklin Lakes, NJ, USA) and incubated under microaerophilic conditions (6 to 16% O_2_ and 2 to 10% CO_2_) using BD GasPak EZ Campy Sachets (Becton Dickinson and Company, Franklin Lakes, NJ, USA) at 48 ± 3 h at 35 ± 1 °C. Enumeration for APC-M, APC-P, Coliforms, and *Escherichia coli* was conducted using the 3M™ Petrifilm Plate Reader (3M™, St. Paul, MN, USA) while for LAB a Q-Counter (Spiral Biotech Inc, Norwood, MA, USA) was used.

### 2.6. Instrumental Color Analysis

Instrumental color measurements were recorded every 12 h on chops displayed in the retail case for a total exhibition period of 96 h. Measurements were obtained in triplicate using a reflectance spectrophotometer (Hunter MiniScan XE, Model 45/O-S; Hunter Associates Laboratory Inc., Reston, VA, USA) equipped with a 6 mm measurement port, calibrated with an Illuminant D65 and 10° standard observer. Comission Internationale de l’Eclairage (CIE) L*, a*, and b* values were recorded and further used to calculate hue angle and chroma.

### 2.7. Visual Color Analysis

Trained color panelists (*n* = 6–8) evaluated lean color, fat color, and percent lean discoloration every 12 h on chops displayed in the retail case for a total exhibition period of 96 h. Panelists used a Qualtrics survey (Qualtrics XM, Seattle, WA, USA) and evaluated chops according to a randomly assigned number. Attributes for each sample were ranked on an electronic ballot with a 100-point continuous line. For lean color, the zero-point anchor was labeled as pale-pinkish-gray to white and 100-point anchor was labeled as dark-purplish-red. For fat color, the zero-point anchor was labeled as tannish-brown, and the 100-point anchor was labeled as bright-white. Finally, for percent discoloration a continuous line rated from 0 to 100 was used.

### 2.8. Statistical Analysis

All data were analyzed using R (Version 4.1.3) statistical analysis software (R Core Team, Vienna, Austria) to evaluate the growth rate of microbial indicators as well as the change rate of instrumental and visual color measurements through retail case display at each of the different dark storage periods. Microbial loads were analyzed at each dark storage period and retail display time combination. Counts were transformed to Log CFU/cm^2^ and a Kruskal–Wallis test was performed comparing the effect of treatment throughout the retail case display time at each dark storage period, followed by a pairwise comparison Wilcoxon’s-test adjusted by the Benjamini and Hochber method. All significant differences were evaluated using a *p*-value lower than 0.05.

Furthermore, microbial counts were transformed into Log CFU/cm^2^ and linear regression analysis was performed with APC-M, APC-P, and LAB-M counts as well as Hue, Chroma, Lean Color, and Percentage Discoloration as dependent variables to obtain the change rate of each of the variables per treatment and unit of time (day). The model contained one qualitative variable (Treatment) with five levels (Water, Bovibrom 225 ppm, Bovibrom 500 ppm, Fit Fresh 3 ppm, Washing Solution 750 ppm) and one continuous variable (retail case display time). The water treatment was used as the base level for the qualitative variable and 95% confidence intervals were created for each of the slopes obtained for each of the microbial indicators and instrumental and visual color measurements for statistical comparisons. As an example, the model for APC-M is described below (Equation (1)).

(1) $$\begin{array}{ll} Log\;Counts= & \beta_{0}+\beta_{1}Trt1+\beta_{2}Trt2+\beta_{3}Trt3+\beta_{4}Trt4+\beta_{5}Time\\ & \;\;+\beta_{6}Trt1^{\prime }Time+\beta_{7}Trt2^{\prime }Time+\beta_{8}Trt3^{\prime }Time\\ & \;\;+\beta_{9}Trt4^{\prime }Time+\varepsilon \end{array}$$

For the model, $\beta_{i}$ are parameters, Trt1 stands for Bovibrom 225 ppm, Trt2 stands for Bovibrom 500 ppm, Trt3 stands for Fit Fresh 3 ppm, Trt4 stands for Washing Solution 750 ppm, Time stands for Retail Display Time (Days), and ε is the error term. The procedure used random effects regression for slopes estimations give the nature of panel data. The model accounted for potential violation of the constant variance.

Principle component analysis (PCA) was completed for instrumental color analysis (L*, a*, b*, Hue, and Chroma), visual color analysis (Lean color, fat color, and % discoloration), and microbiological analysis (APC-M, APC-P, LAB-M, and Coliforms) data separately using PROC FACTOR in SAS (Version 9.4; SAS Inst., Cary, NC, USA). Two principal components (PC1 and PC2) were established and prior to PCA all data was mean centered and standardized.

## 3. Results and Discussion

For pork loin chops, enumeration of mesophilic and psychrotrophic aerobic plate counts and lactic acid bacteria were performed at 0 h, 48 h, and 96 h after the end of each of the four dark storage periods (Day 1, Day 14, Day 28 and Day 42) at refrigerated temperatures 0–4 °C on a retail case.

**Figure 1:**
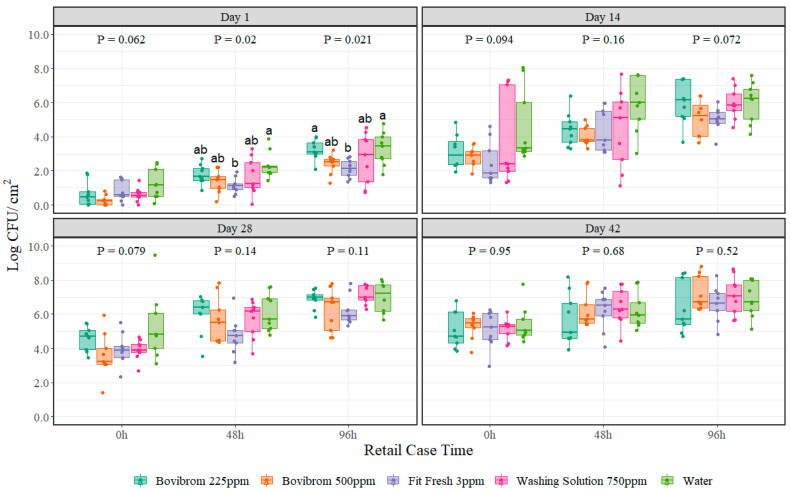
Mesophilic aerobic plate counts (Log CFU/cm^2^) on pork sections treated with different antimicrobials stored at four different dark storage periods (Day 1, 14, 28, and 42) and then cut into chops and retail displayed for 96 h at refrigerated conditions (*n* = 9 swabs per treatment/dark storage period/ retail display time). In each boxplot, the horizontal line crossing the box represents the median, the bottom and top box are the lower and upper quartiles, the vertical top line represents 1.5 times the interquartile range, and the vertical bottom line represents 1.5 times the lower interquartile range. ^(a–b)^ For each dark storage period and retail display time combination, boxes with different letters are significantly different according to Kruskal–Wallis analysis followed by a Wilcoxon’s pairwise comparison test at *p* < 0.05. The points represent the actual data points.

For mesophilic aerobic plate counts, no interaction between treatment and retail display time was found (*p* = 0.19) for any of the four dark storage periods. As the main effect retail display time was found to be significant for all four dark storage periods (*p* < 0.001), a comparison between treatments for each dark storage period and retail display time was performed. Bacterial cells multiply over time; reason why the biological importance lies in the effect of the treatments over time. Moreover, a non-parametric approach test was used to analyze the data as these types of tests do not assume that the data follow any specific distribution. Results obtained in this experiment neither follow a normal distribution nor the sample size is big enough, reason why a Kruskal–Wallis test was used instead of ANOVA to find differences between treatments on each of the retail case display times tested. Differences on mesophilic aerobic plate counts were found only at 48 h (*p* = 0.02) and 96 h (*p* = 0.02) of retail display time at Day 1 of dark storage period between treatments (Figure 1). Treatment Fit Fresh 3 ppm was significantly different at both retail display times (48 h and 96 h) when compared with Water. Moreover, by looking at the *p*-values on each dark storage period, there is an increase in the numerical value throughout time, suggesting a greater similarity among the means of the treatments when compared with Water.

The efficacy of a treatment is highly dependent on the physical and chemical properties of the active ingredient of the antimicrobial [29]. Some of them can affect the permeability of the cytoplasmic membrane (Washing Solution 750 ppm = rhamnolipid) [30]; others can interfere in cell metabolism by oxidation of essential compounds for bacterial enzymes (Bovibrom 225 and 500 ppm = 1,3-dibromo-5,5-dymenthylhydantoin [DBDMH]) [31,32,33]; and others can affect both, cell metabolism and integrity of cell membrane (Fit Fresh 3 ppm = chlorine dioxide) [29,34]. The main effect of using antimicrobials in shelf-life studies is to extend the lag phase of the bacterial cycle, thus delaying their growth, and according to the literature and professional’s experience, a meat product around 6 Log CFU/cm^2^ of aerobic counts is considered to be spoiled even though other attributes shall be taken into account for establishing shelf-life [35]. In this experiment, reaching the 6-log mesophilic APC population limit is dependent on the dark storage and retail display time as at Day 14 of dark storage the limit is reached by 96 h of retail display time, while at Day 28 the limit is reached at 48 h for the majority of the treatments (Figure 1). These results are just guidelines for pork producers and retail stores, as the shelf life of this product will fluctuate depending on the conditions found in grocery or convenience stores. Normally, these products do not stay more than 14 days at dark storage before being displayed in a retail case.

**Figure 2:**
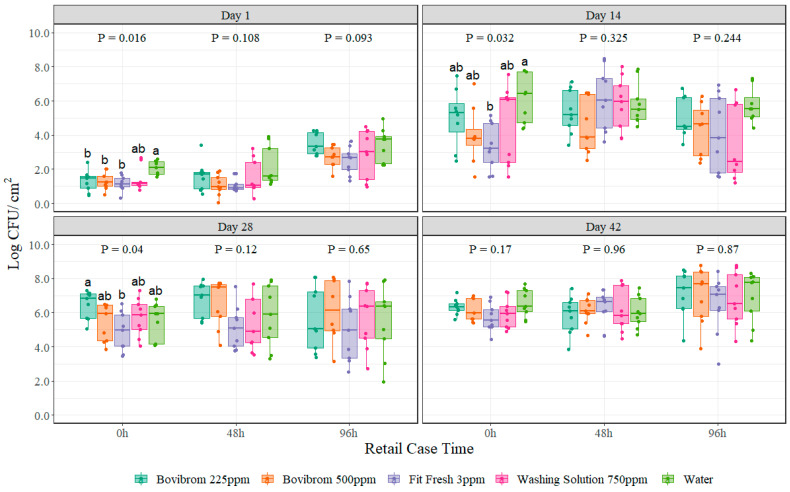
Psychrotrophic aerobic plate counts (Log CFU/cm^2^) on pork sections treated with different antimicrobials stored at four different dark storage periods (Day 1, 14, 28, and 42) and then cut into chops and retail displayed for 96 h at refrigerated conditions (*n* = 9 swabs per treatment/dark storage period/ retail display time). In each boxplot, the horizontal line crossing the box represents the median, the bottom and top box are the lower and upper quartiles, the vertical top line represents 1.5 times the interquartile range, and the vertical bottom line represents 1.5 times the lower interquartile range. ^(a–b)^ For each dark storage period and retail display time combination, boxes with different letters are significantly different according to Kruskal–Wallis analysis followed by a Wilcoxon’s pairwise comparison test at *p* < 0.05. The points represent the actual data points.

For psychrotrophic aerobic plate counts, no interaction between treatment and retail display time was found (*p* = 0.39) for any of the four dark storage periods. Treatments Bovibrom 225 ppm, Bovibrom 500 ppm, Fit Fresh 3 ppm were statistically different when compared with Water (*p =* 0.01) at 0 h display time for Day 1 of dark storage period (Figure 2). Only treatment Fit Fresh 3 ppm was significantly different when compared with Water (*p* = 0.03) at 0 h display time for Day 14 of dark storage period. Furthermore, the variability shown by the boxplots for psychrotrophic aerobic plate counts is greater when compared with mesophilic aerobic plate counts.

The aerobic plate counts are intended to indicate the level of a microorganism in a product and it is the most widely used indicator for quality tests [36]. Mesophilic aerobic plate counts demonstrate an overall microbial load of a sample, however in products that are mostly stored under refrigerated conditions during production, transportation, processing, and post-purchase, psychrotrophic aerobic plate counts represents a more accurate count of bacteria, thus a better indicator for quality [37]. This can be clearly seen in the results obtained during this experiment with similar initial counts between mesophilic and psychrotrophic aerobic bacteria at the beginning of the trial, but throughout extended periods of storage, psychrotrophic microorganism reached higher levels close to 0.5–1.0 log CFU greater compared with mesophilic aerobic counts. Similar results were observed on beef subprimals subjected to spray and dry chilling over prolonged refrigerated storage [37]. Moreover, regarding at the distribution of the dots inside the boxplots, in mesophilic aerobic counts there is always an increase in counts for all dark storage periods throughout retail display time, while for psychrotrophic aerobic counts at Day 28 that increase is less pronounced. Refrigerated and no-oxygen conditions during dark storage may extend the lag phase of mesophilic aerobic counts causing an increase in counts throughout time, however, psychrotrophic bacteria are able to start their log phase and multiply faster thus, reaching stable counts (stationary phase) quicker, too [38]. Results demonstrate that for all treatments similar counts are obtained for Day 28 and Day 42 at all retail display times (Figure 2).

**Figure 3:**
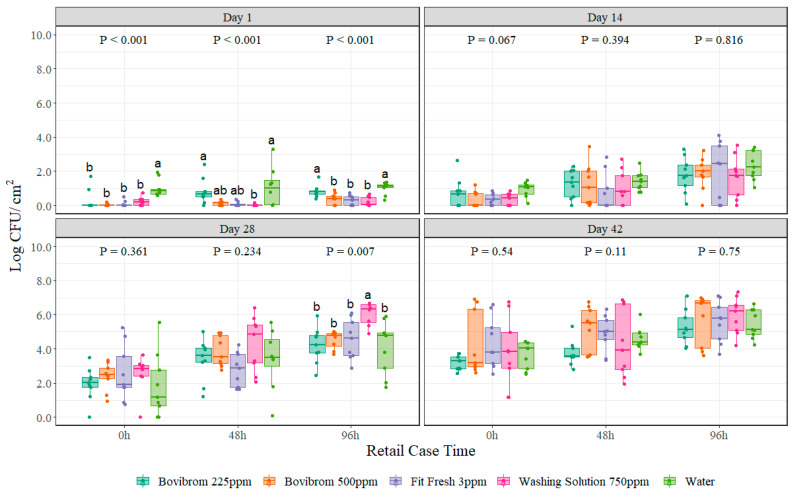
Lactic acid bacteria counts (Log CFU/cm^2^) on pork sections treated with different antimicrobials stored at four different dark storage periods (Day 1, 14, 28, and 42) and then cut into chops and retail displayed for 96 h at refrigerated conditions (*n* = 9 swabs per treatment/dark storage period/ retail display time). In each boxplot, the horizontal line crossing the box represents the median, the bottom and top box are the lower and upper quartiles, the vertical top line represents 1.5 times the interquartile range, and the vertical bottom line represents 1.5 times the lower interquartile range. ^(a–b)^ For each dark storage period and retail display time combination, boxes with different letters are significantly different according to Kruskal–Wallis analysis followed by a Wilcoxon’s pairwise comparison test at *p* < 0.05. The points represent the actual data points.

For lactic acid bacteria, no interaction between treatment and retail display time was found (*p* = 0.38) for any of the four dark storage periods. All treatments were different from Water (*p* < 0.001) at 0 h of retail display time at Day 1 of dark storage, and treatments Bovibrom 500 ppm, Fit Fresh 3 ppm and Washing Solution 750 ppm were significantly different (*p* < 0.001) from Water at 96 h of retail display time at Day 1 of dark storage (Figure 3). Only treatment Washing Solution 750 ppm was different from Water at 96 h on Day 28 (*p* = 0.007).

Lactic acid bacteria is a group of Gram-positive, nonsporing cocci or rods identified as the major spoilage bacteria in vacuum packaged fresh and processed meat stored at room temperature [39]. The combination of the micro-aerophilic conditions, pH around 5.5 to 5.8, presence of curing salt and reduced water activity favors the growth of psychrotrophic lactic acid bacteria [40]. Lactic acid bacteria are found to be the predominant group isolated from vacuum packaged meat products including *Lactobacillus sakei*, *Leuconostoc carnosum*, and *Lactobacillus curvatus* [40]. During logarithmic and stationary growth phase, organoleptic changes become noticeable. Lactic acid bacteria produce lactic and acetic acid, which results in the production of slime and off-odors [41]. In this experiment, comparing aerobic plate counts with lactic acid bacteria counts clearly shows how a big proportion of the total bacteria found in the sample is predominantly lactic acid bacteria, clearly explained by the storage conditions of the pork section before retail display and the presence of sour off-flavors, swelling of the pack and greenish color seen during sampling in this experiment [39].

**Figure 4:**
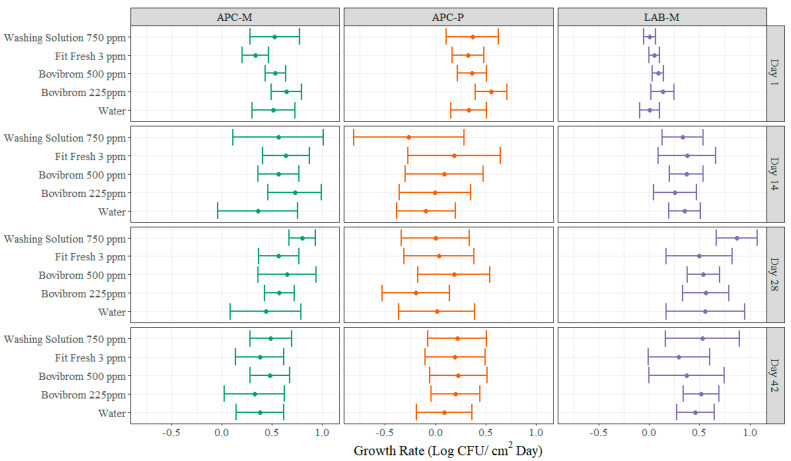
Average (±95% confidence intervals) growth rates (Log CFU/cm^2^ × Day) for each microbial indicator (APC-M, APC-P, and LAB-M) and treatment (Water, Bovibrom 225 ppm, Bovibrom 500 ppm, Fit Fresh 3 ppm, and Washing Solution 750 ppm) at each dark storage period (Day 1, 14, 28, and 42). The dot represents the average growth rate and the horizontal lines represent the 95% confidence interval.

**Figure 5:**
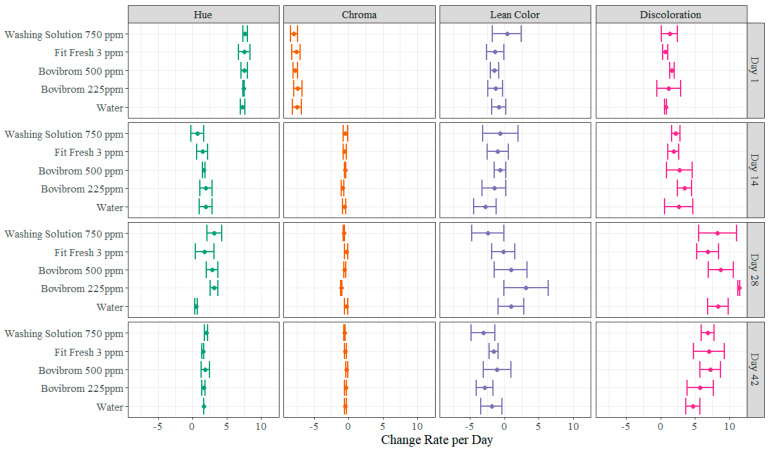
Average (±95% confidence intervals) change rate per day for instrumental and visual color measurements (Hue, Chroma, Lean Color, and Percentage Discoloration) and treatment (Water, Bovibrom 225 ppm, Bovibrom 500 ppm, Fit Fresh 3 ppm, and Washing Solution 750 ppm) at each dark storage period (Day 1, 14, 28, and 42). The dot represents the average change rate of each attribute and the horizontal lines represent the 95% confidence interval.

A novel approach for statistical analysis of shelf-life studies is also presented in this experiment (Figure 4 and Figure 5). Common statistical approach on shelf studies consists in two-way ANOVA analysis including “time” as one factor and “treatment” as the remaining factor. Each factor may include different levels or more than two factors can be included. Interactions or main effects significance are evaluated and pairwise comparison between the combination of time × treatment are normally presented to find differences between treatments at specific sampling points [30,42,43,44]. Other approaches when multiple variables are measured for determining shelf-life, which is common in these type of experiments, are multivariate analysis, specifically the most common one is principal component analysis, which is a statistical method that allow researchers to identify the most important directions of variability and present data in a more understandable way as graphical plots for this type of analysis are very intuitive [21]. Normally, in shelf life studies the question to be answered is, “what is the shelf-life of a certain product?”, meaning what is the maximum allowable time in which the food will remain safe and may retain all their sensorial and functional characteristics, when stored under the specific study conditions [45]. In order to answer that question, multiple factors need to be considered such as microbial load, odor, color measurements and overall appearance of the product, as the shelf-life should be established at the moment when any of these factors fail.

For this approach, multiple linear regressions models were created for each treatment (Water, Bovibrom 225 ppm, Bovibrom 500 ppm, Fit Fresh 3 ppm, and Washing Solution 750 ppm) at each dark storage period (Day 1, Day 14, Day 28, and Day 42) for all different microorganisms and instrumental and color visual measurements. An example will be discussed for a better understanding of the methodology using APC-M as dependent variable at Day 1 of dark storage period. Linear regressions were created using retail display time as continuous variable and treatment as discrete variable with Water as the base level (Equations (2)–(6)).

(2) $$Log\;Counts=\beta_{0}+\beta_{5}Time$$

(3) $$Log\;Counts=\beta_{0}+\beta_{1}Trt1+(\beta_{5}+\beta_{6})Time$$

(4) $$Log\;Counts=\beta_{0}+\beta_{2}Trt2+(\beta_{5}+\beta_{7})Time$$

(5) $$Log\;Counts=\beta_{0}+\beta_{3}Trt3+(\beta_{5}+\beta_{8})Time$$

(6) $$Log\;Counts=\beta_{0}+\beta_{4}Trt4+(\beta_{5}+\beta_{9})Time$$

The slope of the linear models denotes the rate of change in the dependent variable for every unit change on the independent variable. The slope for the Water treatment is 0.514, suggesting that for every increase in one day of retail display time, there is an increase of 0.514 Log CFU/cm^2^ of APC-M after Day 1 of dark storage (Table A1). Moreover, the slope for Fit Fresh 3 ppm (Trt3) is 0.333, suggesting that for every increase in one day of retail display time, there is an increase of 0.333 Log CFU/cm^2^ of APC-M after Day 1 of dark storage, which is lower than the slope for the Water treatment (Table A1). Then, after extracting all the slopes, 95% confidence intervals can be created for statistical comparison. Some advantages of analyzing shelf-life data by slopes comparison is the possibility of prediction of failure in certain attributes by establishing a threshold value and using the equation to predict when this value will be reached. Furthermore, the intercept of the equation denotes the value that the dependent variable takes when the independent variable is equal to zero. This can be translated to the initial microbial load of the sample before starting the retail display time.

The same analysis can be done for instrumental and color visual measurements. As an example, for percentage discoloration the slope for Fit Fresh 3 ppm is lower when compared with Water treatment for Day 1, 14, and 28 of dark storage period. The slope for Day 14 for the Water treatment is 2.6472, suggesting that there is an increase of 2.6472% in percentage discoloration for every increase in one day of retail display time (Table A2). On the other hand, for treatment Fit Fresh 3 ppm the slope is 1.8958, suggesting that for every increase in one day of retail display time, there is an increase in percentage discoloration of 1.8958% (Table A2). In general, after Day 14 of dark storage the hue angle and chroma change rates were almost insignificant between treatments, while during Day 1 of dark storage, both measurements were significantly affected with greater hue angle changes and lower chroma changes. Furthermore, lean color and percentage discoloration in general the change rate was consistent throughout dark storage.

**Figure 6:**
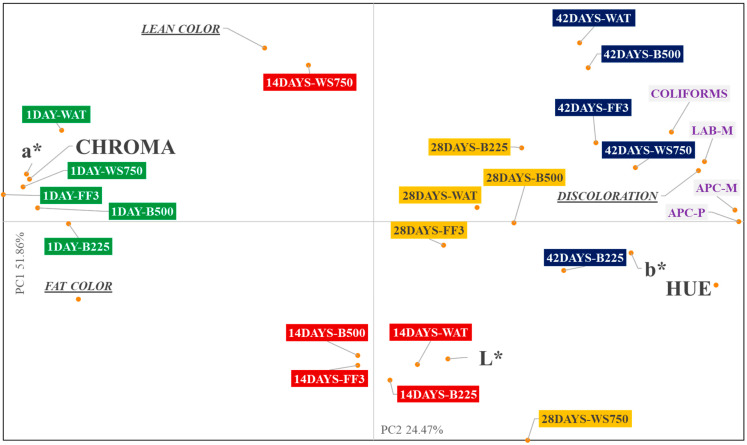
Bi-plot of principal component analysis (PCA) for all microbial and color attributes measured for shelf-life study.

Principal component analysis (PCA) was completed for all treatments and dark storage time combinations (Figure 6). For the PCA, PC1 explained 51.86% and PC2 24.47% of the variation associated with instrumental color analysis, visual color analysis, and microbiological analysis of all dark storage time x treatment combinations. PC1 separated treatments according to dark storage time. Treatments combinations with one day of storage time where clustered together with values of a* and chroma, while treatments combination with 28 and 42 days of storage time where clustered with values of b*, hue, % discoloration and bacteria counts (APC-M, APC-P, LAB-M, and Coliforms). Additionally, L* were clustered with treatment combinations at 14 days of storage time.

These results suggested that pork with less dark storage time followed by retail display presented better red color and highest color vividness, while pork with longer dark storage time followed by retail display were more related with higher yellow colors, higher percentages of discoloration and higher bacteria counts. Deoxymyoglobin is the chemical state of myoglobin where no oxygen is bounded to the sixth coordinate of the heme iron resulting in purple meat colors, meanwhile, oxymyoglobin is the chemical state where the sixth coordinate is occupied by oxygen providing a cherry-red color to meat [46]. During the first days of dark storage time and retail display, pork chops presented greater red color as showed by a* values, lower values in hue angle, and high values in chroma, suggesting that in the first days of storage there was a high presence of oxymyoglobin in pork chops. Reaching the final days of dark storage time and retail display, pork chops presented lower a* values, higher hue values, and lower values for chroma, suggesting that in the last days of storage time there were a lower presence of oxymyoglobin in pork chops and a transition from red color to brown color. Sulfmyoglobin is a chemical state of myoglobin related with bacteria, as the production of hydrogen sulphide converts the muscle pigment to a greener color which is closely related with the decrease of a* values [46]. Research on pork loin chops have found that unaged loins had lower b* and higher a* values when compared to chops in a simulated retail display for 1 to 3 days [47]. In another study, evaluating the Longissimus dorsi at 1 and 8 days of vacuum storage and then displayed in retail up to 6 days, a* values increased markedly during the first day of display but decreased at reaching longer display times for both aging times, whereas the b* only increased after 1 day of aging and then maintained until reaching the longer display time [48].

Aging effect has an impact on blooming, as aging decrease mitochondrial oxygen consumption leading to an increase in bloom intensity [48]. Aging may cause a decrease in mitochondrial content because of possible generation of reactive oxygen species that can cause mitochondrial degradation, explaining the reduction in oxygen consumption, thus improving blooming for the lack of competition for oxygen with myoglobin [49]. On the other hand, aging increased microbial counts in pork, promoting the discoloration, loss of vividness, loss of redness and possible formation of sulfmyoglobin or choleomyoglobin which are related with green colors [50]. The reduction in metmyoglobin is crucial to meat color life and it can occur via two ways: (1) mitochondrial electron transport chain or (2) enzymatic through NADH-dependent metmyoglobin reductase [49]. The reduction in oxymyoglobin is a two-step process in which first, oxymyoglobin is converted to metmyoglobin and then, due to the muscle reducing capacity, this metmyoglobin can be converted to a deoxymyoglobin [25]. This reduction process is dependent on the ability of the mitochondria and the muscle reduce activity to both consume oxygen. Additionally, metmyoglobin color can be formed as there are low oxygen transmission rates due to surface contaminations as aerobic bacteria use oxygen. This suggests that at longer dark storage time, where there is less mitochondrial amount, less NADH available and more bacteria reducing the oxygen transmission rate, the formation of metmyoglobin is more rapid, relating this to the high percentage discoloration founded by panelists and hue angle.

Relation between b*, hue angle and bacteria counts suggest that measures would be useful indicators of percentage discoloration perceived for panelists. Colors represented by b* (blue and yellow) are not commonly related to meat, however, some sensory evaluation studies have found that b* was more correlated to brown as described by sensory panelists, than blue or yellow colors, which partly supported the claim presented in this study [25]. Our results also point that the perception of lean color was not related with any of the instrumental attributes measured, meanwhile, other studies suggest that the degree of lightness and the balance between red and yellow color (hue angle) are highly related with the visual perception done by the panelists [27].

## 4. Conclusions

The purpose of the study was to determine the shelf life of pork loins with the application of different antimicrobials evaluating microbial growth and sensory characteristics. For the microbial analysis, the antimicrobials Bovibrom 500 ppm, Fit Fresh 3 ppm and washing solution 750 ppm performed the best maintaining reduced counts below 6 logs until 42 days of storage time for pork loin chops. For the color analysis, the treatment Fit Fresh performed the best on chops, with great color stability during the whole storage time. In general, the treatment Fit Fresh 3 ppm slowed down the growth of bacteria and maintained the stability of the color, thus extending the shelf-life of pork loins.

## Data Availability

The data are available from the corresponding author.

## Appendix A

**Table A1:** Slopes and 95% Confidence Intervals for Mesophilic Aerobic Plate Counts (Log CFU/cm^2^ × Day).

Dark Storage Time	Treatment	Slope (Log CFU/cm^2^ × Day)	Lower 95% C.I. (Log CFU/cm^2^ × Day)	Upper 95% C.I. (Log CFU/cm^2^ × Day)
Day 1	Water	0.5139	0.3020	0.7258
	Bovibrom 225 ppm	0.6431	0.4932	0.7929
	Bovibrom 500 ppm	0.5347	0.4333	0.6362
	Fit Fresh 3 ppm	0.3325	0.2019	0.4631
	Washing Solution 750 ppm	0.5275	0.2800	0.7750
Day 14	Water	0.3603	−0.0374	0.7580
	Bovibrom 225 ppm	0.7267	0.4592	0.9942
	Bovibrom 500 ppm	0.5630	0.3596	0.7665
	Fit Fresh 3 ppm	0.6394	0.4083	0.8706
	Washing Solution 750 ppm	0.5625	0.1133	1.0117
Day 28	Water	0.4371	0.0834	0.7908
	Bovibrom 225 ppm	0.5725	0.4262	0.7188
	Bovibrom 500 ppm	0.6489	0.3581	0.9397
	Fit Fresh 3 ppm	0.5661	0.3646	0.7676
	Washing Solution 750 ppm	0.8000	0.6704	0.9296
Day 42	Water	0.3794	0.1415	0.6174
	Bovibrom 225 ppm	0.3256	0.0287	0.6224
	Bovibrom 500 ppm	0.4769	0.2794	0.6745
	Fit Fresh 3 ppm	0.3792	0.1391	0.6192
	Washing Solution 750 ppm	0.4867	0.2789	0.6944

C.I.: Confidence intervals.

**Table A2:** Slopes and 95% Confidence Intervals for Percentage Discoloration.

Dark Storage Time	Treatment	Slope	Lower 95% C.I.	Upper 95% C.I.
Day 1	Water	0.7505	0.5886	0.9124
	Bovibrom 225 ppm	1.1924	−0.5448	2.9297
	Bovibrom 500 ppm	1.6668	1.3663	1.9672
	Fit Fresh 3 ppm	0.6920	0.3556	1.0284
	Washing Solution 750 ppm	1.3334	0.1673	2.4994
Day 14	Water	2.6472	0.6080	4.6865
	Bovibrom 225 ppm	3.5069	2.4990	4.5149
	Bovibrom 500 ppm	2.7528	0.8849	4.6207
	Fit Fresh 3 ppm	1.8958	1.1018	2.6899
	Washing Solution 750 ppm	2.2222	1.6129	2.8315
Day 28	Water	8.3524	6.8488	9.8559
	Bovibrom 225 ppm	11.4308	11.2803	11.5814
	Bovibrom 500 ppm	8.7790	6.9911	10.5670
	Fit Fresh 3 ppm	6.8855	5.2768	8.4942
	Washing Solution 750 ppm	8.2907	5.5553	11.0262
Day 42	Water	4.6714	3.6404	5.7023
	Bovibrom 225 ppm	5.7856	3.8927	7.6786
	Bovibrom 500 ppm	7.2156	5.7373	8.6939
	Fit Fresh 3 ppm	7.0408	4.7917	9.2900
	Washing Solution 750 ppm	6.9077	5.9727	7.8428

C.I.: Confidence interval.
